# Hydrogel-Encapsulated Mesoporous Silica-Coated Gold Nanoshells for Smart Drug Delivery

**DOI:** 10.3390/ijms20143422

**Published:** 2019-07-12

**Authors:** Bo Sang Kim, Yi-Ting Chen, Pannaree Srinoi, Maria D. Marquez, T. Randall Lee

**Affiliations:** Department of Chemistry and the Texas Center for Superconductivity, University of Houston, Houston, TX 77204, USA

**Keywords:** drug delivery, temperature-responsive, gold nanoshell, hydrogel coating

## Abstract

A “smart” core@shell composite nanoparticle (NP) having dual-response mechanisms (i.e., temperature and light) was synthesized, and its efficacy in the loading and release of small molecules was explored. These core@shell NPs are composed of an optically active gold nanoshell (GNS) core and a mesoporous (*m*-) silica layer (*m*-SiO_2_). The GNS@*m*-SiO_2_ nanoparticles are further encapsulated within a thermo-responsive poly(N-isopropylacrylamide-co-acrylic acid) hydrogel (PNIPAM-co-AA). The multi-responsive composite NPs were designed to create thermally and optically modulated drug-delivery vehicles with a *m*-SiO_2_ layer providing additional non-collapsible space for drug storage. The influence of the *m*-SiO_2_ layer on the efficacy of loading and release of methylene blue, which serves as a model for a small-molecule therapeutic drug, was evaluated. The “smart” core@shell composite NPs having a *m*-SiO_2_ layer demonstrated an improved capacity to load and release small molecules compared to the corresponding NPs with no *m*-SiO_2_ shell. Additionally, an efficient response by the composite NPs was successfully induced by the thermal energy generated from the gold nanoshell core upon exposure to near infrared (NIR) stimulation.

## 1. Introduction

Motivation for the synthesis of metal nanoparticles (NPs) and their composites has been driven in recent years by the unique and tunable optical properties of these materials and their potential utility in various applications. However, simple metal nanoparticles offer limited utility in many practical applications due to the narrow breadth of their optical properties. This drawback has led to the exploration of alternative nanoparticle configurations, particularly core@shell geometries. Among the many core@shell composite particles, those that respond to stimulation from the core particle (e.g., optically-responsive or magnetically-responsive cores) and the shell through an independent or correlated external stimulus (e.g., temperature, ionic strength, or pH) have garnered interest. Numerous studies have reported their fabrication and potential applications in controlled drug delivery [1,2], chemical catalysis [3,4], and electronics [5,6]. By integrating multiple components—the physical and chemical properties of both materials in composite core@shell materials—multifunctional devices that enable a variety of advanced applications that cannot be accomplished by simple nanoparticles alone can be realized [7,8,9,10,11]. An example of such an application is a targeted photothermal drug-delivery system (DDS).

For biological applications such as DDSs, core@shell composite materials with a dielectric core, such as silica (SiO_2_)—optically transparent and easy to synthesize/modify—and a biocompatible gold shell prove beneficial [12,13]. The unique optical properties that contribute to a metal nanoparticle’s strong interaction with light originate from the collective oscillation of conducting electrons near their surfaces and is referred to as the “surface plasmon resonance” (SPR). The optical properties of nanoparticles can be precisely tuned to maximize the absorption/scattering of light [10,14]. Of particular interest to researchers are gold nanoshells and the ability to tune their extinction into the near infrared (NIR) region (~700-1200 nm) by varying the thickness of the gold shell and the size of the dielectric core [11,15]. Interestingly, NIR light can penetrate human tissue and skin without causing damage, giving rise to the term known as the “water window” for these wavelengths [16]. Access to the NIR spectral region is useful for applications such as medical therapeutics [13], photonics [17], catalysis [18], and biological assays [19]. Most importantly, the absorption of light by these nanoshells leads to rapid and efficient heating, which can be combined with a biocompatible layer to load drugs and produce a nanoscale drug-delivery system that can be specifically targeted and optically activated [20,21,22,23,24].

Another aspect that needs consideration for the development of a targeted photothermally-exposed drug-delivery system is the selection of an optimal shell material. The shell material should be one that is biocompatible and have the capability of acting as a “smart” delivery vehicle. The drug-carrying capacity of *m*-SiO_2_ arises in part due to a large pore volume in the etched silica surface, a feature that is tunable to achieve a specific mesopore diameter in the range of 2-50 nm [25,26]. However, *m*-SiO_2_ NPs by themselves are not “smart” materials because these NPs cannot release drugs in a precise and controlled manner at a specific location (i.e., they have irreversible pore openings). To overcome this drawback, the growth of a stimuli-responsive hydrogel polymer coating on the *m*-SiO_2_ layer can be incorporated due to its ability to release model drugs upon the collapse of the hydrogel matrix [27,28].

Hydrogel polymers have proven to be popular materials for a variety of technological applications such as drug-delivery systems, chemical separations, and catalysis [3,4,29,30]. Hydrogels in aqueous solution undergo phase-volume transitions derived from a lower critical solution temperature (LCST) along with other chemical or physical environmental factors such as pH, light, as well as enzymatic and redox reactions [31,32,33]. A commonly used thermo-responsive hydrogel polymer is poly(N-isopropylacrylamide), PNIPAM, due to its swelling capacity below the LCST and its ability to dehydrate and shrink above its LCST [30,34]. However, PNIPAM homopolymer hydrogels are limited in their biological uses due to their low LCST of ~30 °C [35]. To raise the LCST of PNIPAM-based polymers, acrylic acid (AA) or acrylic amide (AAm) have been copolymerized into the hydrogel polymer matrix, a modification that provides a means of raising the LCST of the hydrogel copolymers anywhere from ~30 °C up to 60 °C [36]. The presence of AA or AAm in the hydrogel backbone can lead not only to an increase in the LCST, but can also cause the copolymer to undergo reversible, discontinuous volume changes that depend on the LCST [36,37].

An ongoing goal of our research is to develop a versatile hydrogel-encapsulated-*m*-SiO_2_-coated NP system that responds to external stimuli to both load and release therapeutic agents. Herein, we present a method for the synthesis of discrete NIR-responsive and temperature-responsive hydrogel-encapsulated *m*-SiO_2_ gold nanoshells (GNS@*m*-SiO_2_@hydrogel) and their use in the loading and release of methylene blue, which serves as a model for a small-molecule therapeutic drug. The hydrogel layer used in our system was a poly(N-isopropylacrylamide)-co-acrylic acid, P(NIPAM-co-AA), consisting of 6 wt% acrylic acid and having an LCST of 34-40 °C. In particular, we wished to use the *m*-SiO_2_ layer to prevent unwanted structural changes to the gold shell during photomodulation and to act as a reservoir for carrying drug payloads to targeted locations. An analogous GNS-based system having no *m*-SiO_2_ layer was also evaluated to evaluate the performance of our composite system. Taken as a whole, this approach of forming composite nanoparticles involving a core nanomaterial coated with a *m*-SiO_2_ layer and encapsulated by a hydrogel modulated by an external stimulant offers a promising new system for nanomedicinal therapeutics.

## 2. Results and Discussion

### 2.1. Synthetic Strategy

**Scheme 1:**
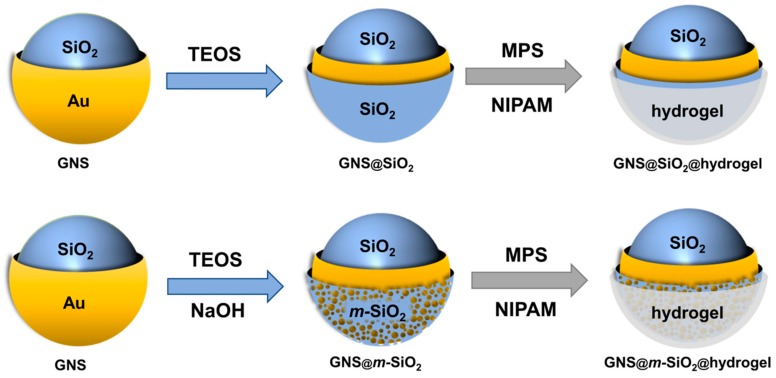
Synthetic strategy used for the synthesis of hydrogel-encapsulated SiO_2_-coated gold nanoshells (GNS@SiO_2_@hydrogel) and hydrogel-encapsulated *m*-SiO_2_-coated gold nanoshells (GNS@*m*-SiO_2_@hydrogel). The hydrogel used was the copolymer poly(N-isopropylacrylamide)-co-acrylic acid (P(NIPAM-co-AA)).

The strategy used to synthesize the GNS@SiO_2_@hydrogel and GNS@*m*-SiO_2_@hydrogel is shown in Scheme 1 and detailed in the Materials and Methods section. Briefly, a modified Stöber method involving polyvinylpyrolidone (PVP) as a surfactant to enhance monodispersity was used to encapsulate gold nanoshells (GNSs) with a silica layer [38,39]. Then, the SiO_2_ coating was further modified through base-etching using a procedure reported by Yin et al. [40]. To coat the *m*-silica layer with the hydrogel copolymer, the surface was modified using 3-(trimethylsilyl) propylmethacrylate (MPS). To encapsulate the composite NPs with the thermo-responsive hydrogel copolymer NIPAM-co-AA, emulsion polymerization was used [41,42,43]. This procedure enables the composite NPs to be stable and chemically resistant, while still being capable of performing their desired task.

Using gold nanoshells as core materials allows for strong optical extinctions that can be precisely tuned anywhere from ~600–1200 nm, giving rise to composite particles that can be activated for drug release using light at NIR wavelengths [44]. Gold nanoshells were reliably grown on silica cores with precise control of the gold shell thickness (~20 nm), producing particles with an absorption maxima at ~800 nm. These nanoshells were coated with silica, then treated with a base solution, detailed in the Materials and Methods section, to produce a porous interface. The P(NIPAM-co-AA) hydrogel was applied to the nanoparticles using emulsion polymerization at 70 °C in an aqueous solution. During the synthesis of the particles, the hydrogel-encapsulating procedure for the *m*-silica-coated nanoparticles was found to be convenient and reliable due to the high solubility of the starting materials under optimized reaction conditions. Moreover, there was no need for purification steps other than filtration. Phase separation in some instances was observed under various conditions, such as reaction time, temperature, or the concentration of the monomers. However, using the procedures outlined above, we found that we could reliably and reproducibly fabricate hydrogel-encapsulated *m*-silica-coated gold nanoshells. Further, the composite particles were found to be colloidally stable, resisting aggregation for more than two months at room temperature.

### 2.2. Characterization of the Composite Nanoparticles

**Figure 1:**
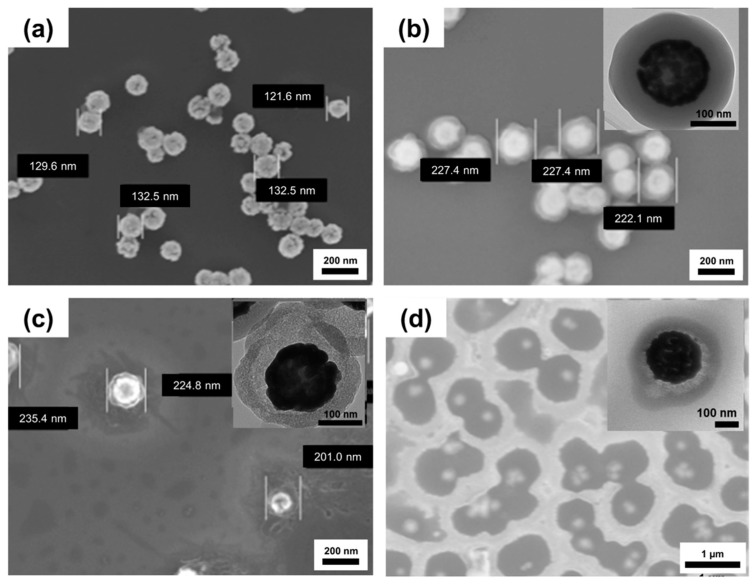
Field emission scanning electron microscope (FE-SEM) images of (**a**) GNSs (140 nm) × 100 K; (**b**) GNS@SiO_2_ (~240 nm) × 100 K; (**c**) GNS@*m*-SiO_2_ (~240 nm) × 100 K; and (**d**) GNS@*m*-SiO_2_@hydrogel (~550 nm) × 30 K. TEM images for the (**b**) GNS@SiO_2_ × 100 K; (**c**) GNS@*m*-SiO_2_ × 100K; and (**d**) GNS@*m*-SiO_2_@hydrogel × 50 K are shown as insets.

Figure 1 shows scanning electron microscope (SEM) images of the gold nanoshells (GNSs), silica-coated gold nanoshells (GNS@SiO_2_), *m*-SiO_2_-coated gold nanoshells (GNS@*m*-SiO_2_), and hydrogel-encapsulated *m*-SiO_2_-coated gold nanoshells (GNS@*m*-SiO_2_@hydrogel), depicting changes associated with each step in the synthesis. The individual images of each step of the synthetic procedure provide a means of determining from one image to the next which part of the composite image is associated with each modification step. The transmission electron microscope (TEM) images confirm the results obtained by scanning electron microscope (SEM) and are shown as insets in Figure 1. The differences in the electron densities of the materials present in the composite NPs allows us to confirm their presence. For example, the silica layer in each of the steps appears as a light contrast layer, the GNS appears as the darkest contrast, while the hydrogel layer as a contrast in between.

Figure 1a shows that the gold nanoshells are homogeneous, due to the monodisperse silica cores (100 nm in diameter), and are ~20 nm thick; similarly, Figure 1b shows that the SiO_2_ coating exhibited a thickness of ~90 nm. Furthermore, etching of the silica shell, to obtain the *m*-SiO_2_, led to no change in the thickness of the shell; the diameter of the particles remained at ~240 nm before (Figure 1b) and after etching (Figure 1c). Furthermore, high magnification TEM images of the silica shell before and after the etching process (Figures S1a and S1b, respectively) show notable changes in the structure of the shell, consistent with the formation of the mesoporous structure. Moreover, the TEM images allow us to evaluate the morphology and thickness of the hydrogel shells individually (i.e., ~150 nm). Thus, we confirm the formation of a hydrogel copolymer layer on the *m*-SiO_2_-coated GNSs from the TEM images. Important to note is the few composite particles with multiple cores encapsulated by a single hydrogel polymer layer generated from the emulsion polymerization, apparent in Figure 1d.

The final composite NPs were analyzed with energy dispersive X-ray spectroscopy (EDX) to confirm their elemental composition (see Figure S2). Analysis by EDX confirms the presence of the key elements in the composite particles, showing characteristic peaks for gold (Mα and Lα at 2.12 and 9.71 keV, respectively), silicon (Kα at 1.75 keV), and carbon (Kα at 0.28 keV). The observation of carbon in the EDX spectra is due to the hydrogel copolymer layer (~150 nm) on the *m*-silica shell. The observation of silicon is either due to the underlying silica core of the gold nanoshell (~20 nm thick), possible defects in the gold shells (e.g., pinholes), the presence of a small number of incomplete gold shells, and/or the *m*-silica layer (~50 nm) on the gold nanoshell. EDX elemental data provide evidence that the key structural components are present for the hydrogel-encapsulated *m*-SiO_2_-coated GNSs. Moreover, the EDX analysis suggests the presence of silica, Au, and the hydrogel copolymer in the final composite nanoparticle and, indirectly supports the hydrogel copolymer as the dominant material on the surface of the composite NPs.

The optical properties of the GNS, GNS@SiO_2_, GNS@*m*-SiO_2_, and GNS@*m*-SiO_2_@hydrogel are provided in Figure S3. Small gold nanoparticles (2–3 nm) are known to have a strong extinction maximum (λ_max_) at 530–570 nm. However, gold nanoshells have been shown to exhibit a broad plasmon band in the 600–1500 nm range [45]. The broadness of the gold nanoshell absorption bands are mostly dependent on the form and fullness of the shell on the silica core [46]. The final GNS@*m*-SiO_2_@hydrogel NPs have a peak position similar to that of the unmodified gold nanoshells due to the thinness of the overlying layers. However, the GNS@*m*-SiO_2_@hydrogel NPs also exhibit a scattering pattern at shorter wavelengths (300–600 nm). We consider the differences in λ_max_ to be minimal among the GNS@*m*-SiO_2_@hydrogel, GNS@SiO_2_, GNS@*m*-SiO_2_, and the bare gold nanoshells. The minimal difference would be due to changes in the refractive index of the hydrogel, *m*-SiO_2_, or SiO_2_ layers which lead to a slight increase in ultraviolet (UV) scattering at shorter wavelengths (300–600 nm), a minor red-shift by the SiO_2_ and hydrogel layers, and a blue shift associated with the *m*-SiO_2_ layer. There is, however, no distinctive shift for λ_max_ for the GNS@*m*-SiO_2_@hydrogel as compared to the bare gold nanoshells.

### 2.3. Thermal Response of the Composite Nanoparticles

The disruption of hydrogen bonds between water and hydrophilic sites within the hydrogel polymer backbone (such as -C=O and -NH) above the LCST has been shown to lead to the breakdown of hydrogel polymer matrices through reduced internal electrostatic repulsion, giving rise to the collapse of the hydrogel polymer layer [47]. Conversely, hydrogen-bond interactions between water molecules and the polymer backbone account for the accumulation of water molecules within the hydrogel network below the LCST and contributes to the electrostatic repulsions that enhance its water-swelling properties [48]. For a pure PNIPAM hydrogel, the LCST has been reported to be ~30 °C. Nonetheless, the LCST can be increased to ~34–40 °C by incorporating AA into the polymer [39,41]. Increasing the LCST allows for the release of small molecules at a temperature close to the body temperature of humans [45,47]. A prior investigation concluded that the increase in the LCST in a copolymer system, similar to the one investigated in this report, occurred due to a decrease in the average inter-chain distance in the hydrogel, leading to an increase in the stability of the H-bonding networks [49]. The increased stability can be attributed to the manipulation of the electrostatic environment by the insertion of ionizable groups, such as AA and AAm, into the polymer backbone.

**Figure 2:**
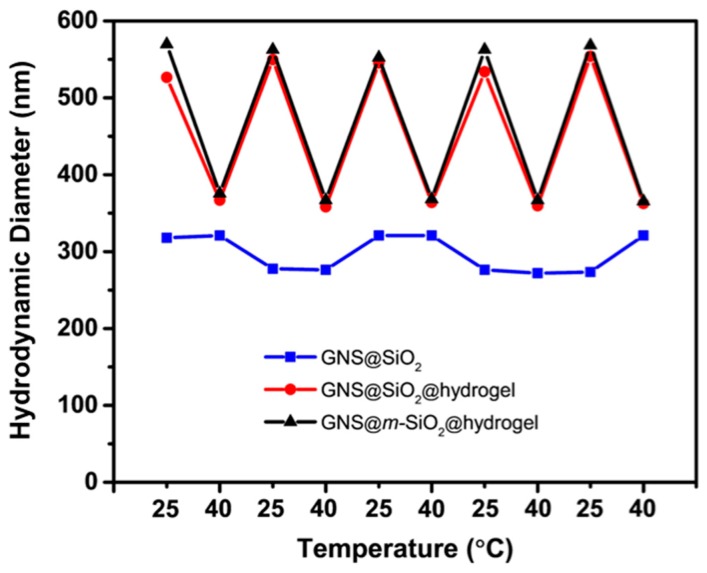
The hydrodynamic diameter of GNS@SiO_2_, GNS@SiO_2_@hydrogel, and GNS@m-SiO_2_@hydrogel, as a function of temperature. The data shown is of a representative set taken from five separate experiments.

To confirm changes in the hydrodynamic diameter of the NPs as a function of temperature, dynamic light scattering (DLS) was used to record the hydrodynamic diameter changes of the GNS@SiO_2_, GNS@SiO_2_@hydrogel, and GNS@*m*-SiO_2_@hydrogel at rt (25 °C) and 40 °C, shown in Figure 2. The diameter of the GNS@SiO_2_ (~300 nm) remains relatively constant with changes in temperature, while the diameter of the GNS@*m*-SiO_2_@hydrogel NPs decrease (~370 nm total diameter) with increasing temperature and increase (~570 nm) with decreasing temperature; a similar trend is also observed with the GNS@SiO_2_@hydrogel NPs (Figure 2). Note, both heating and cooling cycles were repeated five times for each set of nanoparticles and showed similar results each time, indirectly indicating the presence of the hydrogel on the GNS-based NPs.

### 2.4. Loading and Release Capacity of the Mesoporous Layer in the Composite Nanoparticles

Light at wavelengths between 700 and 1200 nm can penetrate human skin without causing any damage [50]. Moreover, the absorption of light by gold nanoshells leads to rapid and efficient heating of the metal shell and the surrounding environment [51]. Furthermore, the nanoshells developed for this research, having a biocompatible *m*-SiO_2_ interlayer and hydrogel layer, can respond rapidly and efficiently to changes in temperature slightly above that found in the body (vide supra). The localized increase in temperature can give rise to the release of small molecules from the hydrogel matrix, providing a targeted drug-delivery system.

**Scheme 2:**
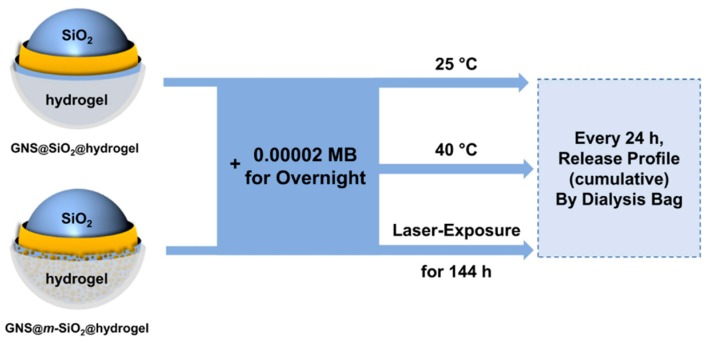
Methylene blue (MB) loading and release experiments.

To evaluate the maximum amount of molecular loading and delivery of the GNS@*m*-SiO_2_@hydrogel at a constant temperature of 25 °C and 40 °C as shown in Scheme 2, we loaded the composite nanoparticles with the organic dye methylene blue (MB) as a model therapeutic agent. To load the hydrogel-coated composite NPs with MB, the particles were placed under vacuum to remove any volatiles, and the resulting dried solid was immersed in a solution of MB in 0.00002 M Tris buffer (pH 7.4) overnight at room temperature. Chemically cross-linked P(NIPAM-co-AA) hydrogel-coated composite NPs have a porous polymer network structure that is particularly suited for trapping small molecules. Moreover, the *m*-SiO_2_ coating provides additional space for carrying small molecules due to their large pore volume (ca. 1 cm^3^/g) and tunable mesopore diameter [25,26]. By mixing the dried composite NPs with a concentrated MB solution, the MB molecules can diffuse into the hydrogel network and mesoporous silica interlayer and become trapped by the hydrogel network.

**Figure 3:**
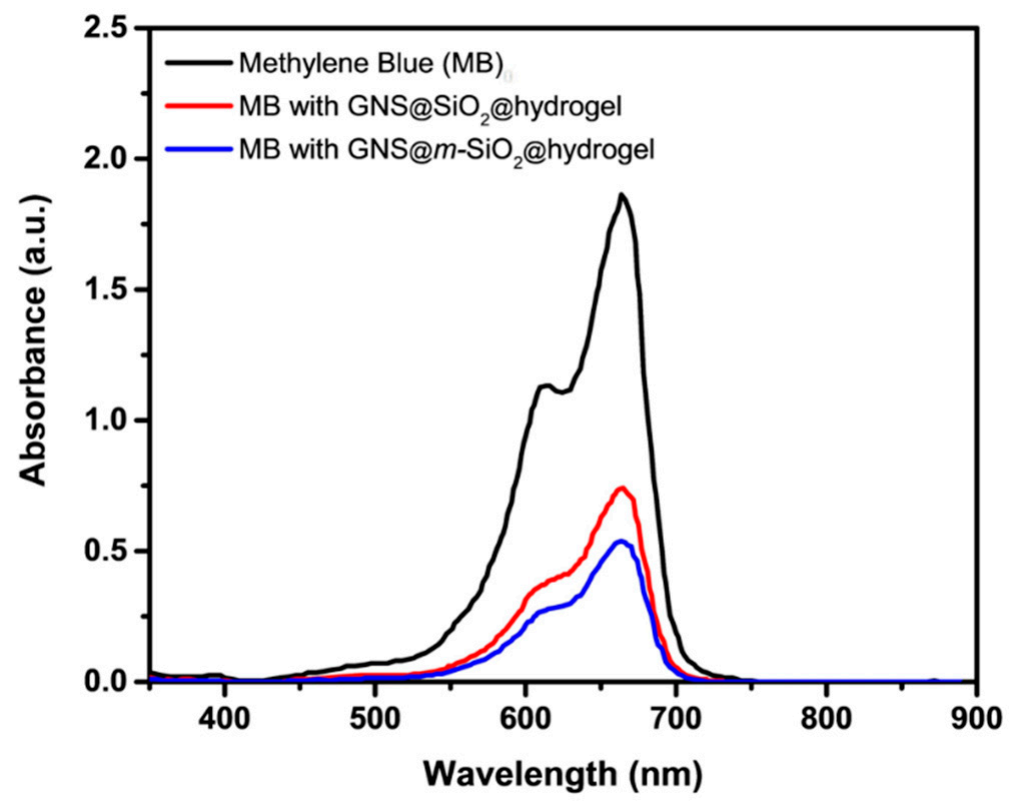
Ultraviolet–visible (UV–vis) spectra of 0.00002 M methylene blue (black) compared to the supernatant of the GNS@SiO_2_@hydrogel (red) and the GNS@*m*-SiO_2_@hydrogel (blue).

We first determined the influence of the mesoporous SiO_2_ on MB loading efficiencies by comparing the GNS@*m*-SiO_2_@hydrogel to the analogous GNS-based composite particle without the mesopores, GNS@SiO_2_@hydrogel. With respect to the concentration of MB, the loading efficiencies were determined to be ~61 % and ~71 %, for the GNS@*m*-SiO_2_@hydrogel and GNS@SiO_2_@hydrogel, respectively, as shown in Figure 3 (please see the Materials and Methods section for details). However, it should be noted that the loading efficiency could be adjusted by varying the amount of composite NPs in the loading solution and/or the concentration of MB. The MB loading efficiency for the GNS@*m*-SiO_2_@hydrogel is ~10 % higher than that for the GNS@SiO_2_@hydrogel due to the higher surface area of the silica shell coming from the large pore volume of the mesoporous silica interlayer [25,26].

**Figure 4:**
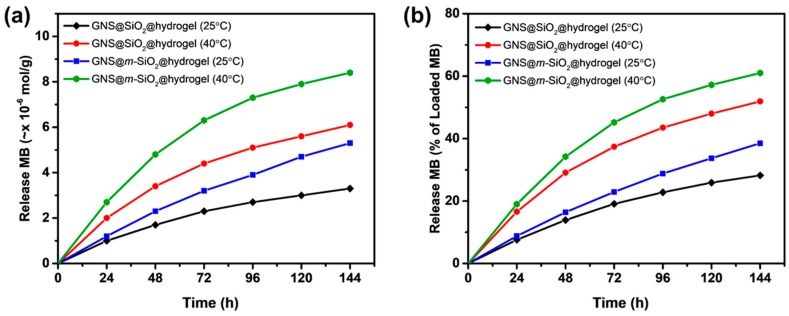
Release profiles of MB-loaded hydrogel-encapsulated composite NPs at constant temperatures of 25 °C and 40 °C; (**a**) released MB in terms of concentration (~× 10^−6^ mol/g) and (**b**) released MB percentage as compared to the loaded MB from hydrogel-encapsulated composite NPs.

After loading, the MB-loaded hydrogel-coated composite NPs were collected and redispersed in Tris buffer to evaluate their release profile. We then maintained our samples at a constant temperature of 40 °C, which is just above the LCST of our P(NIPAM-co-AA) hydrogel. The samples were analyzed every 24 h for 144 h. At the end of each 24 h timeframe, the MB-loaded hydrogel-coated composite NPs were centrifuged, and the concentration of the released MB in the supernatant was determined by UV-vis spectroscopy (as described in the Materials and Methods section). As a control, we performed the same experiment at a constant temperature of 25 °C, which is ~10 °C lower than the LCST. The cumulative release of MB was evaluated at selected intervals of time, and the collected data are shown in Figure 4. For both trials, the release profiles began to plateau by the end of the experimental procedure (see Scheme 2). A much faster release of MB was observed in the release profile at 40 °C for the hydrogel-encapsulated composite NPs; ~30% more MB was released from the hydrogel-coated composite NPs at a constant temperature of 40 °C as compared to the 25 °C control. Interestingly, ~11% more MB was released from the GNS@*m*-SiO_2_@hydrogel at a constant temperature of 40 °C as compared to the GNS@SiO_2_@hydrogel at 40 °C, as shown in Figure 4b.

### 2.5. Photothermal Release Behavior of the Mesoporous Nanoparticles

**Figure 5:**
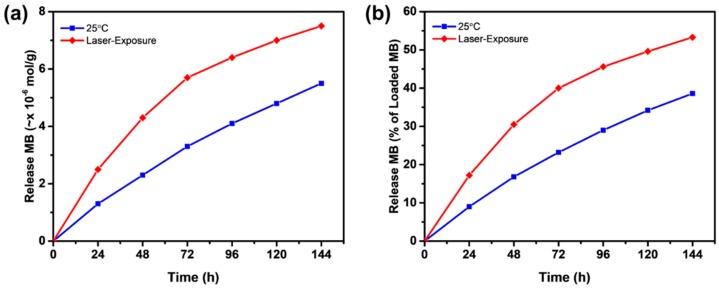
Release profile of MB-loaded GNS@*m*-SiO_2_@hydrogel NPs at a constant temperature of 25 °C and after exposure to a near infrared (NIR) laser; (**a**) released MB in terms of concentration (~× 10^−6^ mol/g) and (**b**) percentage of released MB in terms of the total amount of loaded MB into the hydrogel-encapsulated composite NPs.

To evaluate the potential efficacy of photothermal release of a drug molecule from the hydrogel-encapsulated composite nanoparticles via external stimulation with a NIR laser, we loaded the GNS@*m*-SiO_2_@hydrogel with MB as described above (see the Materials and Methods section for details). The composite NPs were exposed to a NIR laser (wavelength = 810 nm) for 144 h. The composite NPs were then centrifuged at selected intervals during the laser exposure protocol (see Scheme 2), and the supernatant was then analyzed to determine the concentration of the released MB present in the samples by UV-vis spectroscopy, as shown in Figure 5. As a control, we performed the same experiment at a constant temperature of 25 °C, which is ~10 °C lower than the LCST of the hydrogel, without exposure of the NP solution to the laser beam. The cumulative release of MB during this second set of experiments was evaluated at the same time intervals (see Scheme 2), and the collected data are shown in Figure 5. A much faster release of MB was observed in the release profile for the GNS@*m*-SiO_2_@hydrogel that had exposure to the NIR laser beam than those without the laser exposure. Overall, these experiments demonstrate that the release of small molecules from GNS@*m*-SiO_2_@hydrogel can be enhanced by both a direct increase in temperature and exposure to a NIR Laser. Furthermore, this study verifies the anticipated performance of the GNS@*m*-SiO_2_@hydrogel as a model drug-delivery system.

## 3. Materials and Methods

### 3.1. Materials

The chemicals used in this study were purchased from the following companies: ethanol, formaldehyde, sodium hydroxide, ammonium persulfate (APS, 98%), ammonium hydroxide (30% NH_3_), nitric acid, and hydrochloric acid were all from EM Science, Hatfield, PA, USA, while potassium carbonate was from Aldrich, St. Louis, MO, USA. 3-(Trimethylsilyl)propylmethacrylate (MPS, 98.0%), *N*-isopropylacrylamide (NIPAM, 99%), acrylic acid (AA, 99.5%), and the cross-linker *N,N*′-methylenebisacrylamide (BIS, 96.0%) were obtained from Acros; tetraethylorthosilicate (TEOS), tetrakis(hydroxymethyl)phosphonium chloride (THPC), polyvinylpyrolidone (PVP, M_w_ ~55,000), and 3-aminopropyltrimethoxysilane (APTMS) were purchased from Aldrich, St. Louis, MO, USA; and, hydrogen tetrachloroaurate(III) hydrate came from Strem, Newburyport, MA, USA. Water was purified to a resistance of 18 MΩ (Academic Milli-Q Water System, Millipore Corporation, Burlington, MA, USA) and filtered using a 0.22 μm filter to remove any impurities. All glassware and equipment used in the experiment were first cleaned in an aqua regia solution (3:1, HCl:HNO_3_), then cleaned in a base bath (saturated KOH in isopropyl alcohol) and lastly, rinsed with Milli-Q water prior to use.

### 3.2. Synthesis of Gold Nanoshells

Spherical silica core particles having average diameters of ~100 nm were prepared using a modified Stöber method [52]. To overcome the weak affinity of silica for gold, the silane coupling agent 3-aminopropyltrimethoxysilane (APTMS) was used as a surface primer to expose primary amines, which have a strong chemical affinity for gold, on the surface [53,54]. More specifically, after preparation of the silica particles, APTMS (0.5 mL) was added dropwise to the vigorously stirred mixture of silica nanoparticles and ethanol [4]. Stirring was continued for 2 h, and the solution was then refluxed for 1 h at 86 °C to promote covalent bonding of the APTMS groups to the surface of the silica nanoparticle [54]. The amine-functionalized silica particles were then centrifuged using a refrigerated centrifuge at 7000 rpm for 30 min and redispersed three times in ethanol.

A reduced gold (THPC gold) solution was prepared by using a modified procedure found in the literature [55,56]. A 1.0 mL aliquot of a 1 M NaOH solution and 2.0 mL of THPC solution (24 µL of 80% THPC in 2 mL of water) were added to 90 mL of Milli-Q water and vigorously stirred for 10 min, and then an aliquot of 1% aqueous HAuCl_4_·H_2_O solution (4 mL) was quickly added. The color of the solution changed from colorless to reddish brown at once. The solution was stored in the refrigerator at 4 °C for at least three days to age. The size of our aged-THPC gold seed particles was ~2–3 nm in diameter. We deposited the aged-THPC gold seed particles onto the silica core particles using a modified method by Westscott et al. [57]. Briefly, the THPC gold seeds were attached to the silica particles by simply mixing 10 mL of the highly concentrated THPC gold solution (5–10 times) with 1 mL of amine-functionalized silica nanoparticles overnight. To remove any unbound THPC gold seeds, the mixture was then centrifuged and redispersed several times in 25 mL of Milli-Q water. The resulting solution was a light red color after the precipitate was redispersed in Milli-Q water.

We prepared an aqueous solution for growing the gold shells (K-gold solution) using K_2_CO_3_ and HAuCl_4_·3H_2_O. An aliquot of K_2_CO_3_ (0.025 g; 1.81 × 10^−4^ mol) was completely dissolved in 100 mL of Milli-Q water by stirring for 10 min, and then 2.0 mL of 1 wt% HAuCl_4_·3H_2_O was added to the mixture. The color of the solution transformed from yellow to colorless within 30–40 min. After preparation, the K-gold solution was kept in the refrigerator at 4 °C for one day under dark conditions. To grow the gold shell on the THPC gold-seeded silica nanoparticles [57], 8 mL of K-gold solution were placed in a 25 mL beaker with a stirring bar, followed by 1.2 mL of THPC-gold-decorated silica nanoparticles. The mixture was stirred for at least 10 min, and then 0.025 mL of formaldehyde (38%) was added to reduce the K-gold solution. The color of the solution changed from clear to blue. The resultant gold nanoshells with ~140 nm diameter (~20 nm gold shell thickness) were purified by centrifugation. The precipitates were redispersed in Milli-Q water. These particles exhibited an optical extinction centered at ~750 nm.

### 3.3. Synthesis of the Mesoporous Silica Shell

Polyvinylpyrolidone (PVP) was employed to induce phase transfer of the nanoshells from water to ethanol and to stabilize the surface of the gold shells to obtain SiO_2_-coated composite nanoparticles [38,39]. The PVP-capped gold nanoshells were redispersed into a mixture of ammonia (3.0 mL), Milli-Q water (6.0 mL), and ethanol (60.0 mL). The resulting solution was stirred for 10 min, after which TEOS (0.3 mL) was added. The mixture was then further stirred overnight at rt to afford SiO_2_-encapsulated gold nanoshells, which were isolated by washing with copious amounts of ethanol before redispersing in water.

The surface of the SiO_2_ layer was turned into a porous structure by using the procedure reported by Yin et al. [40]. More specifically, the colloidal solution was mixed with PVP (2 g) and brought to reflux for 3 h. After cooling the solution to rt, 15 mL of NaOH solution (0.08 g/mL) was injected into the mixture to initiate the etching process, followed by vigorous stirring for 2 h. After several washings, the *m*-SiO_2_ coated particles were suspended in Milli-Q water.

### 3.4. Synthesis of the Hydrogel Layer

The hydrogel-encapsulated *m*-SiO_2_-coated nanoparticles were prepared by an emulsion polymerization procedure in an aqueous solution. To afford the hydrogel encapsulation, the surfaces of the *m*-SiO_2_-coated particles GNS were initially functionalized with vinyl groups. To achieve this, a suspension of the *m*-SiO_2_-coated particle was mixed with MPS (0.20 mL) under vigorous stirring. The reaction mixture was stirred for 24 h at room temperature (rt). Subsequently, the temperature was elevated to 85 °C in order to improve the bond affinity between MPS and the *m*-SiO_2_ shell. The resultant particles were separated and washed with ethanol several times before redispersing in 10.0 mL of ethanol. An aqueous solution containing NIPAM, AA, and BIS, was mixed with the MPS-modified *m*-SiO_2_-coated NP ethanolic solution (5 mL) and placed in a three-necked round-bottomed flask equipped with a reflux condenser and an inlet for argon gas. The solution was degassed with argon for 1 h to remove any oxygen in the flask. This step was performed to reduce the presence of oxygen in the solution, because oxygen can intercept radicals and disrupt the polymerization process. NIPAM (0.2 g; 2 × 10^−3^ mol), AA (10 µL; 1.5 × 10^−5^ mol), and the cross-linker BIS (0.02 g; 1 × 10^−4^ mol) were added to the mixture and stirred for 15 min. The mixture was then heated to 70 °C in an oil bath followed by addition of the initiator APS (0.02 g; 9 × 10^−5^ mol) to induce the polymerization. The reaction was allowed to proceed for 7~8 h followed by cooling the solution to rt. The final particles were washed with deionized water and filtered with a 1 μm membrane to remove any micron-sized impurities and/or any aggregated particles at the end of the reaction. The filtered solution was centrifuged at 30 °C for 1 h at 5000 rpm two times, and the supernatant was separated to remove any unreacted materials and soluble side products. The purified nanoparticles were then diluted with Milli-Q water and stored at rt for later analysis.

The size of the GNS@*m*-SiO_2_@hydrogel NPs and GNS@SiO_2_@hydrogel NPs were ~550 nm in diameter, a size that was produced by controlling the amount of monomer, the amount of initiator, and the reaction time. A thicker coating of the hydrogel on the gold nanoshells was also achieved by increasing the concentration of the monomers, but this thicker coating was less reproducible than the thin coating. We also observed the formation of multiple *m*-SiO_2_-coated gold nanoshell cores within a single hydrogel shell in the thick hydrogel-coated samples. Therefore, we chose to focus on analyzing the hydrogel-coated gold nanoshells having ~550 nm diameters.

### 3.5. Loading and Release of Methylene Blue (MB)

To load the MB dye inside the hydrogel of the hydrogel-coated nanoparticles, a dye loading procedure was developed and is described as follows using the GNS@SiO_2_@hydrogel NPs as a representative example. A 20 mL aliquot of the GNS@SiO_2_@hydrogel solution was placed under vacuum to form a white powder followed by addition of 5 mL of a solution of 0.00002 M MB in Tris buffer. After stirring overnight at rt, the MB-loaded GNS@SiO_2_@hydrogel NPs were separated from the excess MB molecules by centrifugation at 5000 rpm for 30 min, 2–3 times. The MB-loaded hydrogel-coated composite NPs were deposited as a residue on the bottom of the centrifuge tube after decanting the solution of excess MB. For quantification of the MB loaded inside the hydrogel-coated composite NPs, the UV-vis absorption spectrum of the supernatant MB solution was recorded. The amount of the excess MB was calculated using calibration curves generated at λ = 664.5 nm from standard solutions prepared at known MB concentrations. By subtracting the calculated MB from the original MB solution, the amount of MB loaded inside the hydrogel was obtained.

For the release study, 5 mL of Tris buffer was added to the centrifuge tube containing the MB-loaded hydrogel-coated composite NPs. The solution was stirred for selected intervals of time separately at 40 °C, at 25 °C, or with NIR laser exposure (wavelength = 810 nm) and then centrifuged as described above. The supernatant was collected by decantation, and the concentration of MB in the supernatant was recorded by UV-vis spectroscopy to calculate the released amount of MB from the hydrogel-coated composite NPs. The procedure was repeated for each time interval (every 24 h for 144 h) to generate the release profiles shown in the Section 2.

### 3.6. Characterization Methods

Analysis by SEM was carried out using a LEO-1525 scanning electron microscope operating at 15 KV. To collect FE-SEM images, synthesized nanoparticles were deposited on a silicon wafer and completely dried at rt overnight prior to analysis. All the samples were examined at magnifications of 10 K, 30 K or 50 K, and 100 K in order to demonstrate the overall uniformity of the particles.

Analysis by TEM was carried out using a JEM-2000 FX transmission electron microscope (JEOL) equipped with elemental analysis by EDX (Link ISIS software series 300, Oxford Instruments, Concord, MA, USA) at an accelerating voltage of 200 kV. All of the TEM samples were deposited on 300 mesh Holey carbon-coated copper grids and dried before examination.

A Cary 50 Scan UV-vis optical spectrometer (Varian) with Cary Win UV software was employed to characterize the optical properties of the THPC gold seed-attached SiO_2_ particles, bare GNS, *m*-SiO_2_-coated GNS and GNS@*m*-SiO_2_@hydrogel NPs. UV-vis spectra of the prepared gold nanoshells were collected by diluting the samples with Milli-Q water, transferring them to a quartz cell, and scanning over a range of wavelengths (250–1000 nm). The GNS@*m*-SiO_2_@hydrogel NPs were analyzed as prepared without dilution. The spectra of distinct batches of composite nanoshells were obtained both before and after coating with the hydrogel copolymer for experimental consistency.

The DLS instrument (ALV-5000 Multiple Tau Digital Correlation) was operated with a light source wavelength of 514.5 nm and a fixed scattering angle of 90° for the hydrodynamic diameter measurement of the particles. After measuring 5 times for a duration of 100 s, the average hydrodynamic diameter was determined.

## 4. Conclusions

This research has revealed a method for the reproducible preparation of gold nanoshells and their encapsulation with a biocompatible *m*-SiO_2_ interlayer along with a hydrogel copolymer outerlayer, designed for enhanced drug delivery. The core gold nanoshells were prepared with a diameter of ~140 nm and an extinction maximum at 800 nm. We directly coated particles with a silica layer (~50 nm), which was then etched into a porous structure. The hydrogel overlayer was grown through emulsion polymerization to give a shell thickness of ~150 nm. The morphology, elemental composition, and physical properties of the composite particles were characterized by SEM, EDX, TEM, DLS, and UV-vis spectroscopy. Collectively, the data support the formation of distinct hydrogel-encapsulated *m*-SiO_2_-coated gold nanoshells. Through DLS analysis, the hydrodynamic diameters of the hydrogel-coated composite nanoparticles were shown to reproducibly decrease with increasing temperature over the range of 25 °C to 40 °C. The temperature-responsive behavior of the GNS@*m*-SiO_2_@hydrogel was demonstrated through DLS measurements, showing a reversible change of the particle diameter with changing temperature. The surface plasmon resonance of the GNS@*m*-SiO_2_@hydrogel exhibited an extinction maximum at ~810 nm, which falls in an important spectral range for biomedical applications. The NIR-responsive behavior of the GNS@*m*-SiO_2_@hydrogel was demonstrated by experiments involving a NIR laser (wavelength = 810 nm), showing that these composite particles, upon excitation by the laser, undergo an increase in temperature and respond accordingly.

Finally, our fabrication method can be applied for use on other nanoparticle systems including various composite structures. The response of the hydrogel-encapsulated GNS@*m*-SiO_2_@hydrogel to light at NIR wavelengths and to changes in temperature are completely consistent with the targeted objectives for their use as drug-delivery vehicles. The GNS@*m*-SiO_2_@hydrogel showed an improved loading capacity as compared to the particles prepared without a porous silica shell and an increase in the numbers of small molecules subsequently released. The rate and amount of the released molecules from these composite NPs could be regulated by adjusting the temperature and the exposure to the NIR laser beam. Given this combination of properties, our GNS@*m*-SiO_2_@hydrogel NPs offer considerable promise for use in targeted photo-initiated drug delivery.

## Supplementary Materials

Supplementary materials can be found at https://www.mdpi.com/1422-0067/20/14/3422/s1. The following are available online: Figure S1: TEM images of (a) GNS@SiO_2_ and (b) GNS@*m*-SiO_2_; Figure S2: EDX spectrum of the GNS@*m*-SiO_2_@hydrogel NPs; Figure S3: UV-vis spectra of (a) GNSs, (b) GNS@SiO_2_, (c) GNS@*m*-SiO_2_, and (d) GNS@*m*-SiO_2_@hydrogel under neutral conditions.
