# Supplementary material for: Hydrogel-Encapsulated Mesoporous Silica-Coated Gold Nanoshells for Smart Drug Delivery
Source: Int J Mol Sci. 2019 Jul 12;20(14):3422. doi: 10.3390/ijms20143422 (PMC6678574; doi:10.3390/ijms20143422)
Supplement: Supplementary file 1 [file ijms-20-03422-s001.pdf]

Supporting Information

for

Hydrogel-Encapsulated Mesoporous Silica-Coated Gold Nanoshells for Smart Drug Delivery

*Bo Sang Kim, Yi-Ting Chen, Pannaree Srinoi, Maria D. Marquez, and T. Randall Lee**

Department of Chemistry and the Texas Center for Superconductivity,
University of Houston, Houston, TX 77204, USA;

k2617799@naver.com (B.S.K.); ychen75@uh.edu (Y.-T.C.); psrinoi@uh.edu (P.S.); mdmarquez2@uh.edu (M.D.M.)

*Correspondence: trlee@uh.edu; Tel.: +1-713-743-2724

Table of Contents:

1. TEM Images of (a) GNS@SiO_2 and (b) GNS@*m*-SiO_2 (Figure S1)
2. Optical Properties of Hybrid Particles (Figure S2)
3. EDX Spectrum of GNS@*m*-SiO_2@hydrogel (Figure S3)

1. TEM Images of GNS@SiO_2 Before and After Etching

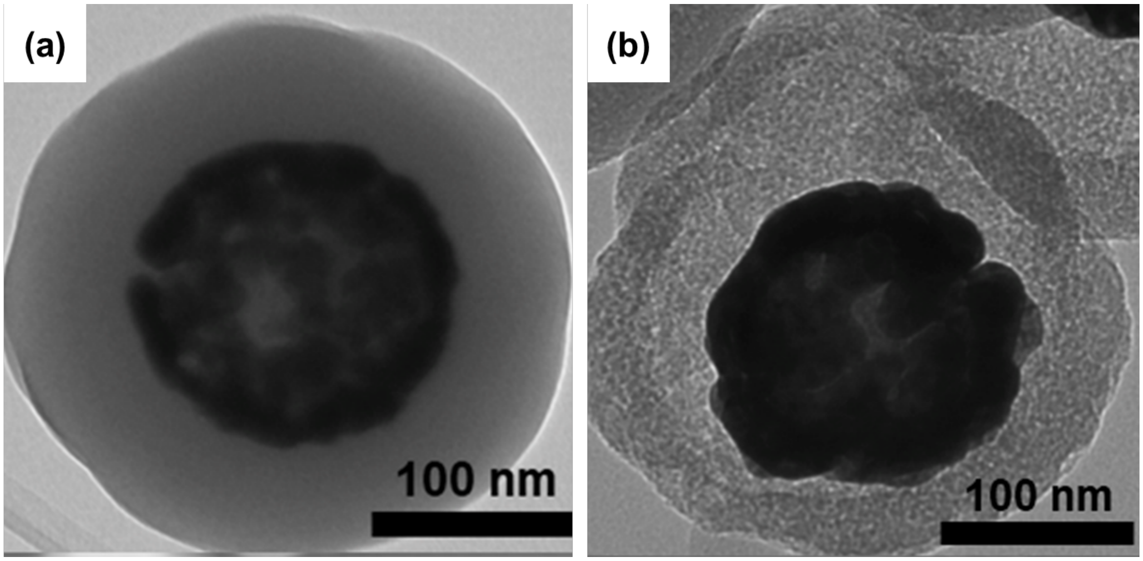

Figure S1. TEM images of (a) GNS@SiO_2 and (b) GNS@*m*-SiO_2.

2. Optical Properties of the Hybrid GNSs

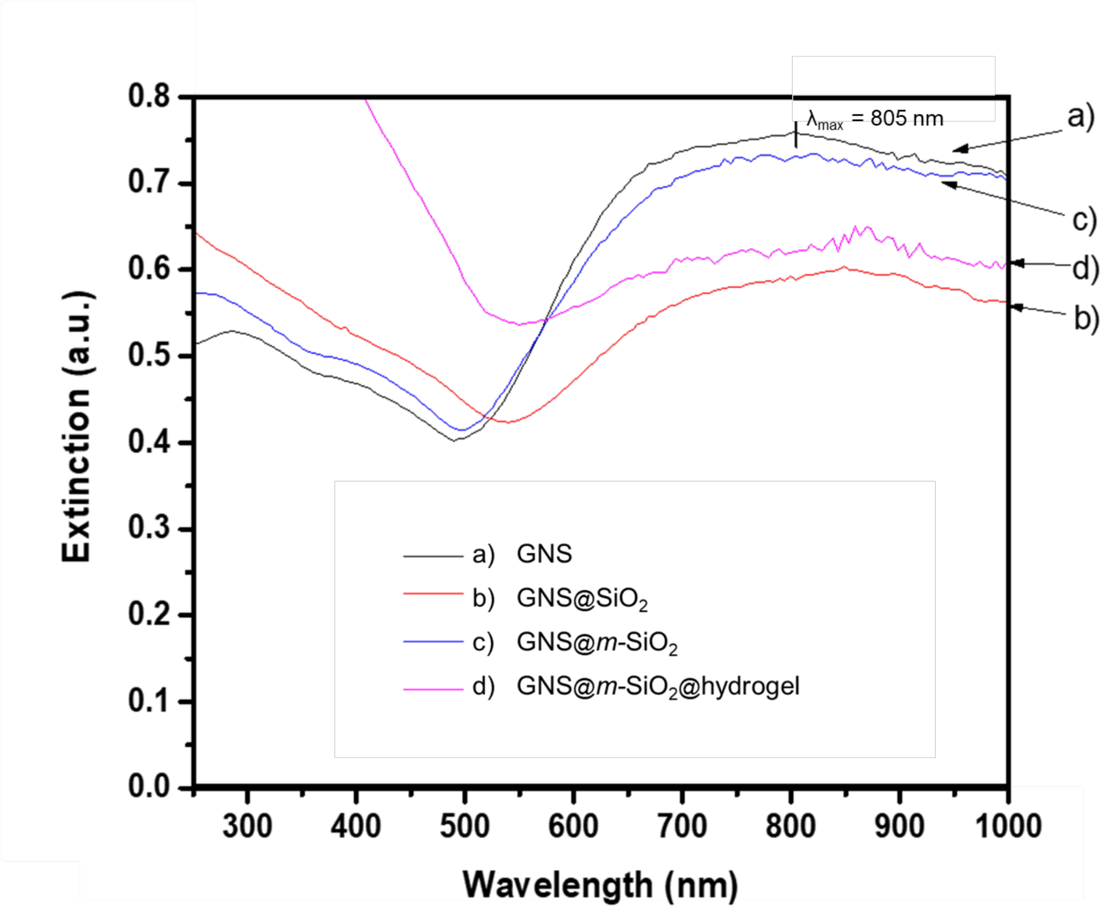

Figure S2. UV-vis spectra of (a) GNSs, (b) GNS@SiO_2, (c) GNS@*m*-SiO_2, and (d) GNS@*m*-SiO_2@hydrogel under neutral conditions.

3. Elemental Analysis of the Composite NPs by Energy-Dispersive X-ray (EDX) Spectroscopy

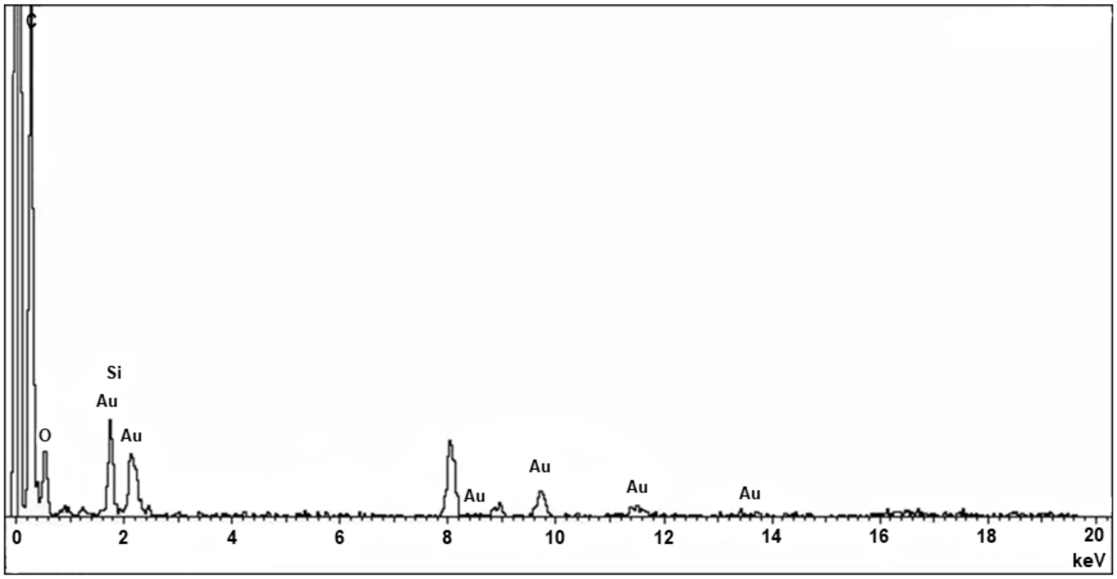

Figure S3. EDX spectrum of GNS@*m*-SiO_2@hydrogel.
